# Microbiological Profile of Adenoid Hypertrophy Correlates to Clinical Diagnosis in Children

**DOI:** 10.1155/2013/629607

**Published:** 2013-09-23

**Authors:** Anita Szalmás, Zoltán Papp, Péter Csomor, József Kónya, István Sziklai, Zoltán Szekanecz, Tamás Karosi

**Affiliations:** ^1^Department of Medical Microbiology, Medical and Health Science Center, University of Debrecen, Nagyerdei Krt. 98, Debrecen 4032, Hungary; ^2^Department of Otolaryngology and Head and Neck Surgery, Medical and Health Science Center, University of Debrecen, Nagyerdei Krt. 98, Debrecen 4032, Hungary; ^3^Department of Rheumatology, Medical and Health Science Center, University of Debrecen, Nagyerdei Krt. 98, Debrecen 4032, Hungary

## Abstract

*Objective*. Adenoid hypertrophy is a common condition in childhood, which may be associated with recurring acute otitis media (RAOM), otitis media with effusion (OME), and obstructive sleep apnea syndrome (OSAS). These different clinical characteristics have some clinical overlap; however, they might be explained by distinct immunologic and infectious profiles and result in various histopathologic findings of adenoid specimens. *Methods*. A total of 59 children with adenoid hypertrophy undergoing adenoidectomy were studied. Three series of identical adenoid specimens were processed to hematoxylin-eosin (H.E.) and Gram staining and to respiratory virus specific real-time PCR, respectively. *Results*. According to the clinical characteristics, patients were recruited into three groups: RAOM (*n* = 25), OME (*n* = 19), and OSAS (*n* = 15). Bacterial biofilms were detected in 21 cases, while at least one of the studied respiratory viruses was detected in 52 specimens. RAOM cases were significantly associated with biofilm existence (*n* = 20, *P* < 0.001). In contrast, OME group was characterized by the absence of bacterial biofilm and by normal mucosa. Showing a statistically significant correlation, all OME cases were positive for human bocavirus (HBoV, *P* < 0.001). *Conclusions*. Bacterial biofilms might contribute to the damage of respiratory epithelium and recurring acute infections resulting in RAOM. In OME cases persisting respiratory viruses, mainly HBoV, can cause subsequent lymphoid hyperplasia leading to ventilation disorders and impaired immunoreactivity of the middle ear cleft.

## 1. Introduction

Adenoid hypertrophy (adenoid vegetation) and associated symptoms of children are a common condition in pediatrics and ENT practice that is responsible for a great amount of medical visits and one of the main reasons for antibiotic treatment and parental leave of work [1]. Nasopharyngeal obstruction caused by adenoid hypertrophy may lead to several other diseases and symptoms such as hyponasality, snoring, obstructive sleep apnea syndrome (OSAS), acute otitis media, otitis media with effusion (OME), middle ear atelectasia, cholesteatoma formation, slow feeding, acute sinusitis, abnormal facial development, and behavioral problems [2–5]. Adenoidectomy with or without tympanocentesis or ventilation tube insertion is a useful surgical intervention to treat hearing problems, obstructive sleep apnea syndrome, and to prevent recurring otitis media, recurring sinusitis, and cholesteatoma formation or middle ear atelectasia [6–8]. In Hungary (population is 10 million), 6–9,000 adenoidectomies have been performed annually in the latest years emphasizing the extent of this condition. The annual number of reported adenoidectomies varies between 15,000 and 50,000 in the United States and between 50,000 and 70,000 in the European Union [7, 8]. In the clinical practice, symptoms and conditions associated with adenoid hypertrophy can be classified into distinct clinical groups as follows: (1) recurring acute otitis media (RAOM), (2) otitis media with effusion (OME), and (3) obstructive sleep apnea syndrome (OSAS) [9–13]. In contrast to potential clinical overlaps, clinical behavior, recurrence rates, and therapeutic responses are absolutely different in these groups [1, 11, 12, 14]. Hypertrophic adenoid (epipharyngeal tonsil) may directly obstruct the ostia of Eustachian tubes predisposing children to OME [1, 2, 10]. However, this mechanical hypothesis is not widely accepted [15, 16]. Recurring and chronic infections of the adenoids without manifesting nasopharyngeal obstruction may also lead to the aforementioned conditions by tubal edema and functional disorders supporting the reservoir theory [1, 9, 17]. 

Diagnosis of adenoid hypertrophy associated with OME is based on the duration of conductive hearing disorder, type-B tympanograms, and positive otoscopic findings, which persists over 12 weeks despite medical treatment [10, 11]. OME is a separate diagnostic entity, although the etiopathogenesis is still unclarified [3, 9, 15, 16]. Several hypotheses have been arisen in the explanation of the pathogenesis of OME, which is currently thought to be an immunologic disease affected by multiple factors [10, 15, 16]. These are persisting respiratory viral infections, immunological dyscrasias, alimentary allergies, allergic rhinitis, bronchial asthma, and Eustachian tube functional disorders [3, 14, 15, 18]. There is increasing evidence that persisting respiratory viruses and impaired immunoreactions may play a promoting role in the pathogenesis of OME associated with adenoid hypertrophy [18–20].

OSAS in childhood is a complex clinical condition that can be affected by several anatomic and pathologic disorders, as well as adenoid hypertrophy, tonsillar hypertrophy, nasal obstruction, laryngeal malformations, macroglossia, maxillofacial malformations, and so forth [12, 13]. As to our current knowledge, pure OSAS cases associated to simple adenoid hypertrophy have not been attributed to immunologic or infectious disorders [12, 13].

RAOM is also a separate clinical entity, which might be associated to the presence of bacterial biofilms on the surface of epipharyngeal mucosa [17, 21, 22]. Biofilm creates a special environment for microbial survival and proliferation that consists of a self-produced, three-dimensional extracellular matrix formed by polysaccharides, proteins, nucleic acids, and water [21, 23, 24]. Microbial biofilms are characterized by extremely high resistance against antibiotics, host immune reactions, and chemical and physical agents [21, 22]. This strong and continuously remodeled physical barrier blocks the diffusion of antibiotics, superoxides, immunoglobulins, and opsonins [21, 25]. Biofilms and consecutive inflammatory reactions might contribute to the epithelial damage and subsequent hyperplasia of the subepithelial layer resulting in adenoid hypertrophy [1, 9, 23, 24]. It has been reported that persisting biofilms in adenoid hypertrophy may be responsible for surgical failures and high recurrence rate of acute suppurative otitis media [3, 8, 10, 15].

This study investigates the presence of bacterial biofilms and respiratory viruses in adenoid specimens obtained from patients with adenoid hypertrophy and associated conditions. Histopathologic findings and respiratory virus specific real-time PCR results were correlated to the clinical characteristics of prospectively formed patient groups.

## 2. Materials and Methods

### 2.1. Patients

A prospective case-control experimental study was performed on adenoid specimens obtained from children with adenoid hypertrophy who underwent adenoidectomy at the Department of Otorhinolaryngology and Head and Neck Surgery, University of Debrecen. Adenoid samples (*n* = 59) were collected between January and May 2012. The patient group consisted of 37 males and 22 females (*n* = 59, mean age = 5.09 years; range = 3–11 years) with the diagnosis of adenoid hypertrophy and associated symptoms such as otitis media with effusion, recurring acute otitis media, and obstructive sleep apnea syndrome. Clinical diagnosis was confirmed by preoperative evaluation of patients' history obtaining heteroanamnesis from parents and by physical and audiologic examinations. During the prospective data collection, patients were recruited into three clinical categories forming distinct groups of (1) recurring acute otitis media (RAOM), (2) otitis media with effusion (OME), and (3) obstructive sleep apnea syndrome (OSAS), respectively. Inclusion criteria were based on the rate of episodes of acute suppurative otitis media, acute sinusitis or sinobronchitis, on the otoscopic findings, audiologic examinations (pure tone threshold audiometry and tympanometry), and finally on patients' history. All surgical interventions were performed under intratracheal narcosis. All the adenoid samples collected during adenoidectomy were divided into two consecutive groups consisting of 59 specimens each that were processed to histopathologic analysis and respiratory virus specific real-time PCR, respectively. All adenoid specimens were larger than 2 centimeters of diameter, which could be removed by a sharp adenotom device without any surface injury or iatrogenic disruption of biofilm layers. The transoral removal was gently performed at the basis of adenoids avoiding the surgical injury of *torus tubarius*, nasal septum, and the mesopharyngeal mucosa. In several cases, adenoidectomy was combined by tympanocentesis and suction of the middle ear fluid content. Middle ear fluids were visually analyzed and categorized into serous or mucous groups. All surgeries were performed by two ENT specialists having more than 10 years of experience (Z. Papp and T. Karosi). All parents gave their informed consent before donating the tissue samples for the study. The Institutional Ethical Committee approved our study. The study was carried out according to the Declaration of Helsinki.

### 2.2. Histopathologic Examination

According to previous reports, combination of H.E. and Gram staining protocols seems to be a usable method for the detection of biofilm existence and corresponding histopathologic changes, since H.E. staining is for the investigation of microscopic architecture, while Gram protocol stains various bacterial elements [25]. The first series of adenoid specimens (*n* = 59) were fixed in 10% (w/v) formaldehyde. Specimens were embedded in 15% (w/v) purified gelatin (24 h, 56°C) and refixed in 4% (w/v) paraformaldehyde (24 h, 20°C). Blocks were cryoprotected in 20% (w/v) sucrose-solution (2 h, 4°C) and sectioned into 5 *μ*m slides at −25°C (MNT-200, Slee, Mainz, Germany). Slides were stored in 0.1 M PBS containing 0.03% (w/v) sodium-azide at 4°C. Two consecutive 5 *μ*m frozen cut sections were examined using conventional staining with hematoxylin and eosin (H.E.) and conventional Gram staining, respectively. The criteria for the histopathologic detection of microbial biofilms were the presence of characteristic morphology and Gram positivity/negativity and microcolonies of bacteria and the presence of the surrounding polysaccharide layer upon examination by optical microscopy [25]. Structure and cellular infiltration of the epithelial and also the subepithelial layers were correlated to the presence of bacterial biofilms. Histologic pretreatment protocols were performed by an independent laboratory assistant in all cases. Both sets of histologic staining protocols were independently analyzed by two researchers (P. Csomor and T. Karosi) blinded to clinical diagnosis.

### 2.3. Nucleic Acid Extraction and Real-Time PCR Detection of Respiratory Viruses

Second series of adenoid specimens (*n* = 59) were processed for nucleic acid extraction and purification, which was performed by High Pure Viral Nucleic Acid Kit (Roche Applied Science, Basel, Switzerland) according to the manufacturer's instructions. Simultaneous detection of respiratory viruses with potential pathogenic role was performed by validated and commercial RT (reverse transcriptase) and real-time PCR systems. The following respiratory viruses were tested: influenza virus A and B (Iv-A and B); respiratory syncytial virus A and B (RSV-A and B), human metapneumovirus A and B (hMPV-A and B); rhino- and enteroviruses (RV/EV); adenoviruses (AdV, 52 serotypes), and human bocavirus (hBoV 1, 2, 3, and 4). All real-time PCR systems were purchased from Argene (influenza A/B r-gene, Ref: 70-040; RSV/hMPV r-gene, Ref: 70-041; RV and EV/Cc r-gene, Ref: 70-042; AdV/hBoV r-gene, Ref: 70-043). Amplification reactions were performed following a uniform protocol according to the manufacturer's specifications and instructions and the results were validated with positive, negative, and cellular controls provided within the amplification kits. Real-time PCR products were electrophoretized in 10% (w/v) agarose gel in all cases.

### 2.4. Statistical Analysis

Statistical assessments were performed by Mann-Whitney's *U* probe with a 95% confidence interval and by Chi-square test (SPSS 9.0 for Windows).

## 3. Results

### 3.1. Patient Groups

Altogether fifty-nine patients with adenoid hypertrophy who underwent adenoidectomy were included in this study. The clinical history and findings of physical and audiologic examinations were obtained during the confirmation of the diagnosis of adenoid hypertrophy and associated problems. Clinical pieces of information on bronchial asthma, allergic rhinitis, and covering symptoms were recorded in different patient groups before surgery (Table 1). 

### 3.2. Recurring Acute Otitis Media (RAOM) Group

For this group of patients, the inclusion criteria were based on 2 or more acute suppurative otitis media and acute sinusitis or sinobronchitis episodes in the year before adenoidectomy (Table 1). Patients suffering from otitis media with effusion confirmed by physical examination and tympanometry were excluded from this group. In contrast, associated obstructive sleep apnea syndrome was not an exclusion criterion. This group of patients consisted of sixteen males and nine females (*n* = 25; mean age 5.12 years; range 3–11 years). The average number of acute suppurative otitis media episodes was 6.72/year (range: 2–14), while the acute sinusitis episodes reached the average annual number of 5.41 (range: 2–9) (Table 1). One patient had allergic rhinitis that was based on the clinical history, on the physical examination, and on the allergen-specific intracutaneous (Prick) skin test. Preoperative systemic antibiotic treatment (amoxicillin, cefuroxim, azithromycin, or clarithromycin) was performed in all patients due to recurring acute upper airway tract infections.

### 3.3. Otitis Media with Effusion (OME) Group

In this patient group, the inclusion criteria were based on persisting middle ear fluid content and Eustachian tube functional disorder confirmed by physical examination and tympanometry (Table 1). Symptoms persisted more than six weeks in spite of medical treatment including intranasal steroids and/or systemic antihistamine therapy. Patients with unilateral or bilateral middle ear effusion were also recruited in this group. Obstructive sleep apnea syndrome was not an exclusion criterion. This group of patients consisted of twelve males and seven females (*n* = 19; mean age 4.63 years; range 3–8 years). The average number of acute suppurative otitis media episodes was 1.21/year (range: 0–4), while the acute sinusitis episodes reached the average annual number of 1.31 (range: 0–3) (Table 1). Nine patients had allergic rhinitis and four patients were diagnosed with bronchial asthma. The diagnosis of bronchial asthma was based on the clinical history and on the respiratory functional test. The final diagnosis of bronchial asthma was stated by an experienced pulmonologist in all cases. Systemic antihistamine medication (cetirizine or desloratadine) and topical steroid (mometasone-furoate monohydrate) treatment were performed in all patients.

### 3.4. Obstructive Sleep Apnea Syndrome (OSAS) Group

In this group of patients, the inclusion criteria were based on the persisting (more than three months) sleep apnea and snoring confirmed by the heteroanamnesis obtained from the parents (Table 1). Patients with several recurring acute suppurative otitis media and acute sinusitis episodes or chronic otitis media with effusion confirmed by physical examination and tympanometry were excluded from this group. This group of patients consisted of nine males and six females (*n* = 15; mean age 4.93 years; range 3–11 years). The average number of acute suppurative otitis media episodes was 0.33/year (range: 0-1), while the acute sinusitis episodes reached the average annual number of 0.4 (range: 0-1) (Table 1). Four patients had allergic rhinitis and two other patients had bronchial asthma.

### 3.5. Real-Time PCR Detection of Respiratory Viruses

**Figure 1:**
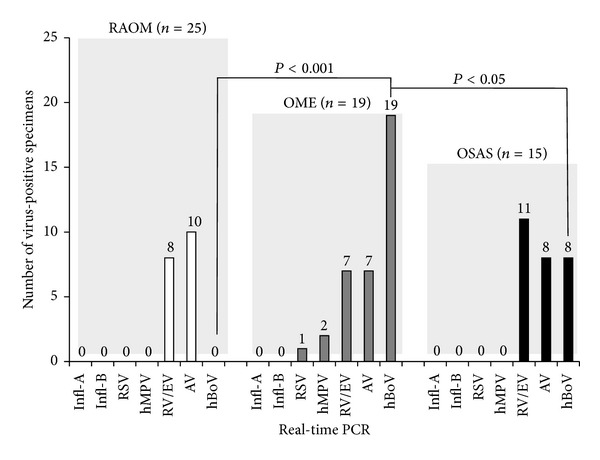
Graphic representation of respiratory virus specific real-time PCR results in different patient groups. RAOM: recurring acute otitis media; OME: otitis media with effusion; OSAS: obstructive sleep apnea syndrome.

According to the validated real-time PCR results, persisting A and B types of influenza virus (Iv-A and B) could not be detected in the adenoid specimens (Tables 2 and 3). The detectability of RSV and hMPV did not achieve a significant level and could be established as an accidental association in different patient groups (Tables 2–4). In contrast, RSV and hMPV were exclusively detected in the OME group. The mainly associated viral pathogens were originated from various serotypes of RV/EV, AdV, and HBoV species (Tables 2–4).  Table 2 presents the individual coexpression patterns of different respiratory viruses. All virus-negative cases (*n* = 7) belonged to the RAOM group, while patients of OME and OSAS groups were all characterized by various coexpressions of respiratory viruses (Tables 3 and 4). The RAOM group was exclusively associated with the presence of RV/EV AdV species (Tables 3 and 4). In the OSAS group, HBoV was detected in 54% of cases, which was mainly coexpressed with RV/EV (20%) and AdV serotypes (27%) (Table 4, Figure 1). Only one case was characterized by individual persistence of HBoV (Table 4). All specimens of the OME group were characterized by HBoV (Tables 3 and 4, Figure 1). Furthermore, eight of nine cases with individual expression of HBoV belonged to the OME group (Table 4). It was found that individual presence (*P* < 0.001, Chi-square test) or codetection (*P* < 0.05, Chi-square test) of HBoV in the adenoid tissue is a strong predictor of OME associated with adenoid hypertrophy (Tables 3 and 4, Figure 1). 

### 3.6. Histopathologic Examination

**Figure 2:**
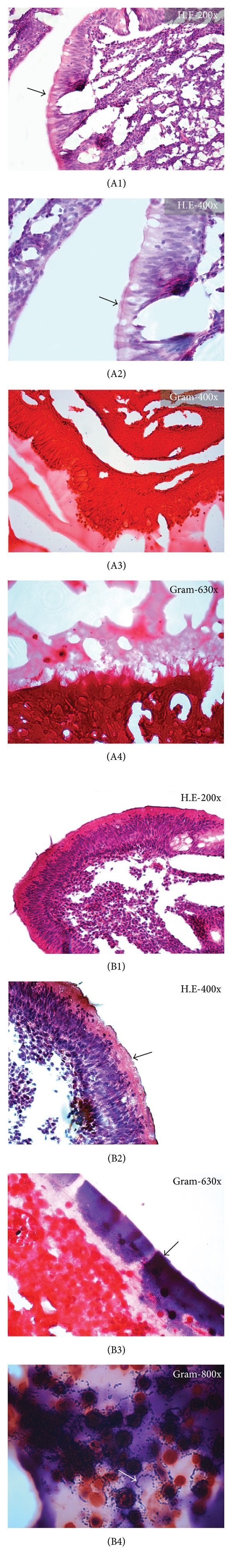
Histopathologic representation of the respiratory epithelium in adenoid specimens of patients from OME and RAOM groups. (A1) At low magnification, regular columnar epithelium can be detected (black arrow, H.E.). (A2) Higher magnification view of the previous section. Black arrow indicates intact ciliated cells. (A3) Biofilm structures cannot be detected on the surface of epipharyngeal epithelium (Gram). (A4) Higher magnification view of the previous section. (B1) The epithelial layer is disintegrated and covered by a thick eosinophilic structure (black arrow, H.E.). (B2) At higher magnification, biofilm layer is a well identifiable structure (black arrow). (B3) Gram staining reveals a bacterial biofilm consisted of individual colonies of Gram-positive cocci (black arrow). (B4) Higher magnification view of the previous section. White arrow indicates Gram-positive cocci in a chain-pattern of streptococci.

**Figure 3:**
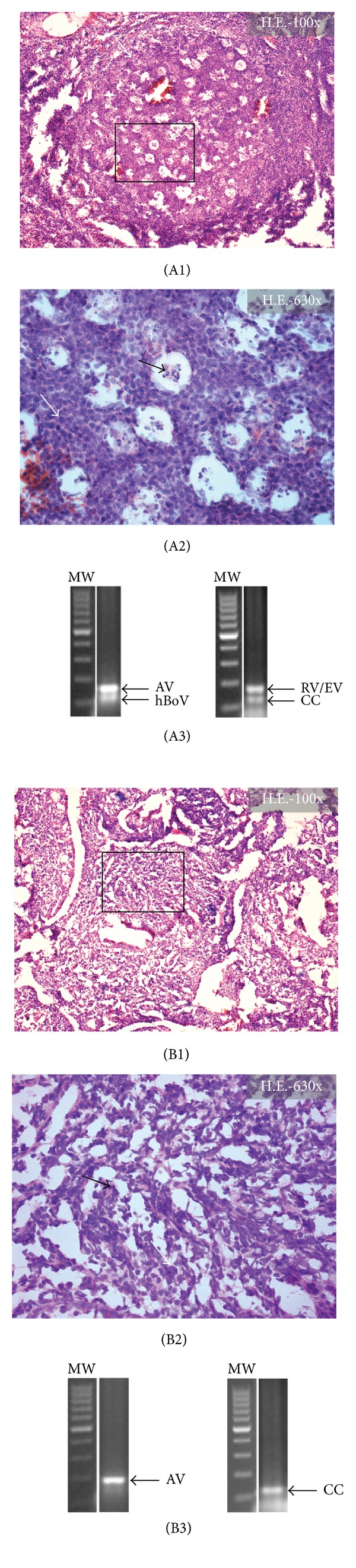
Histopathologic representation of the stromal substance in adenoid specimens of patients from the OME and RAOM groups. (A1) Normal centrum germinativum (white arrow, H.E.). (A2) Higher magnification of the previous section. White arrow indicates large lymphocytes, while immunoblasts and centroblasts are shown by black arrow. (A3) Agarose gel electrophoresis of real-time PCR products of the identical specimen. Positive reactions were shown for Adenovirus (AdV), Human Bocavirus (hBoV), and for Rhinovirus-Enterovirus (RV/EV) serotypes. CC: cellular control. (B1) Disintegrated stromal substance without centrum germinativums (white arrow, H.E.). (B2) At higher magnification view, several polymorphonuclear cells (black arrow) and plasmocytes (white arrow) can be identified. (B3) Agarose gel electrophoresis of real-time PCR products of the identical specimen. The sample showed individual expression of Adenovirus (AV). CC: cellular control.

Histopathologic analysis revealed inflammatory adenoid tissues with lymphocytic, plasmacytic, or eosinophilic and polymorphonuclear infiltration of the subepithelial stroma in all cases. Bacterial biofilms were detected in 21 (35.59%) of 59 patients with adenoid hypertrophy (Table 3, Figure 2). In the biofilm-negative cases (*n* = 38, 64.41%), histopathologic analysis revealed a regular respiratory mucosa with predominantly lymphocytic infiltration of the subepithelial layer (Table 3, Figure 3). Overall, the great majority (>95%) of biofilm-positive cases were originated from the RAOM group, where the prevalence of bacterial biofilms was 80% (Table 3). In contrast, biofilms could not be detected in the OME group. In the OSAS group, bacterial biofilms represented 6% of prevalence (Table 3). Independently from the associated symptoms, all patients diagnosed with allergic rhinitis were recruited into the biofilm-negative group (Table 3). The presence of bacterial biofilms was strongly associated to the histopathologic characteristics of epipharyngeal mucosa and also to the dominant inflammatory cell type of the subepithelial layer (Chi-square test; Table 3, Figures 2 and 3). Biofilm-negative cases were characterized by normal respiratory mucosa with columnar ciliated epithelium and goblet cells (Table 3, Figure 2). Disintegration or cellular metaplasia of the respiratory mucosa was strongly associated to the presence of biofilms (Chi-square-test; Table 3, Figure 2). It was found that polymorphonuclear or plasmacytic infiltrations and disintegrated architecture of the subepithelial (stromal) layer are the most important predictors of biofilm presence (Table 3, Figure 3). In contrast, predominant lymphocytic infiltration and the presence of several centrum germinativum significantly decreased the chance of biofilm detection (Chi-square-test; Table 3, Figure 3). 

## 4. Discussion

In the present study, we demonstrated the presence of bacterial biofilms in 21 patients with adenoid hypertrophy by the combined application of H.E. and Gram staining. Individual presence or coinfections of respiratory viruses were detected in 52 adenoid specimens. Results were correlated to the integrity of respiratory mucosa and also to the microscopic architecture of the subepithelial layer. It was found that the presence of bacterial biofilms or persisting respiratory viruses, mainly HBoV, is a strong predictor of RAOM and OME, respectively. RAOM cases were significantly associated with biofilm existence, epithelial destruction, and stromal disintegration. Furthermore, all virus-negative cases belonged to this group of patients. In contrast, OME cases were characterized by biofilm negativity and normal architecture of the respiratory mucosa and the subepithelial layer. Beyond that all OME cases were positive for HBoV; all individual expressions of this virus were recruited in this patient group. It should be established that the presence of HBoV displayed a statistically significant correlation to OME cases associated with adenoid hypertrophy. The main limitation of our study is the relatively low number of subjects, which should be increased in the future in order to obtain more precise statistical correlations. Nevertheless, we could not exclude the seasonal pattern of respiratory virus infections in our patients. Furthermore, biofilm detection was based on the combined applications of H.E. and Gram staining protocols that were not correlated to the widely accepted methods of biofilm detection such as FISH (fluorescent in situ hybridization), CLSM (confocal laser scanning microscopy), and SEM (scanning electron microscopy) [25]. We did not intend to make a precise identification of bacterial species, because the presence of biofilm itself is thought to be the most important factor in the pathogenesis of OME and RAOM cases [3, 9–11]. 

The mechanical hypothesis emphasizes the importance of nasopharyngeal obstruction caused by adenoid hypertrophy [1, 7]. This theory can explain only a limited fraction of symptoms associated with adenoid hypertrophy, such as nasal obstruction, hyponasality, olfactory dysfunction, snoring, mouth breathing, and disturbed facial and teeth development [1, 5, 12, 13]. It has been reported that physical disproportion between the volume of adenoid and epipharyngeal space is the most important factor in the pathogenesis of OSAS [4, 5, 12]. Beyond the serious pathophysiological consequences, OSAS may cause various behavioral problems, such as anxiety, depression and attention deficits, or hyperactivity [4, 5]. 

The reservoir hypothesis emphasizes the direct or indirect role of persisting respiratory pathogens in the pathogenesis of RAOM and OME associated with adenoid hypertrophy [9, 15, 17, 21]. The main bacterial pathogens in RAOM cases are the *Streptococcus pneumoniae*, *Moraxella catarrhalis*, and *Haemophilus influenzae* [22–24, 26]. Both streptococci and Haemophilus species are potent biofilm-forming pathogens that may result in chronic persistence in the adenoid lacunae [21, 23, 26]. Acute exacerbations of otitis media may originate from this bacterial reservoir due to ascending reinfections via the Eustachian tube [1, 9, 17]. These bacteria can usually be cultured from the purulent effusion obtained from the middle ear [24, 26]. 

In contrast, OME is a chronic disease that is characterized by conductive hearing loss, persisting type-B tympanograms, special otoscopic findings, painless aural fullness, and by mental problems in some cases [3, 6, 10, 11]. The serous or mucous effusions obtained during tympanocentesis or ventilation tube insertion are often negative for bacteria according to the results of conventional microbiological analysis [3, 9, 15, 16]. At this time, OME is suspected to be a complex immunologic disorder of the middle ear cleft, which is mediated by the interactions of persisting respiratory viruses and impaired immune responses [15, 16]. It has been reported that RV/EV species, HBoV, RSV, and AdV might play an important role in the pathogenesis of OME [18, 27–30]. Suggesting the importance of B-cell mediated immunoreaction, Rezes et al. have reported increased albumin-globulin ratio (>0.7) in the middle ear fluids obtained from patients with OME [20]. Recent studies have demonstrated increased expression of IL-4, IL-11, and IFN-gamma and decreased levels of IL-1, TNF-alpha, or TGF-beta in the serous or mucous middle ear effusions suggesting the role of humoral immune response in the pathogenesis of OME [14–16, 20]. 

According to our results, distinct clinical pictures of adenoid hypertrophy can be characterized by different histopathologic and virus expression profiles. Adenoidectomy with or without tympanocentesis or ventilation tube insertion still remains the gold standard for the treatment of adenoid hypertrophy; however, further research may introduce targeted therapies in the future. In conclusion, adenoid hypertrophy-associated conditions are diseases of multifactorial agents involving disturbed local immune response and chronic inflammation. Furthermore, persisting respiratory viruses and bacterial biofilms might contribute to the damage of respiratory epithelium and subsequent hyperplasia of the subepithelial layer leading to functional disorder of the Eustachian tube and impaired immunoreactivity of the middle ear cleft. 

## Figures and Tables

**Table 1:** Clinical characteristics of different patient groups.

Patient groups (n = 59)	Age (years)	Male-female ratio	AOM^1^/year	AS^2^/year	OSAS^3^	Type of middle ear effusion^4^ (number of ears)		Preoperative tympanometry^5^ (number of ears)			Bronchial asthma	Allergic rhinitis
						Serous	Mucous	A	B	C		
RAOM^6^ (*n* = 25)	5.12 (3–11)	16/9 (1.77)	6.72 (2–14)	5.41 (2–9)	2 (8%)	0 (0%)	0 (0%)	31 (62%)	0 (0%)	19 (38%)	0 (0%)	1 (4%)
OME^7^ (*n* = 19)	4.63 (3–8)	12/7 (1.71)	1.21 (0–4)	1.31 (0–3)	17 (89%)	18 (47%)	14 (37%)	5 (13%)	32 (84%)	1 (3%)	4 (21%)	9 (47%)
OSAS^8^ (*n* = 15)	4.93 (3–11)	9/6 (1.5)	0.33 (0-1)	0.4 (0-1)	15 (100%)	0 (0%)	0 (0%)	27 (90%)	0 (0%)	3 (10%)	2 (13%)	4 (27%)

^1^Acute suppurative otitis media. ^2^Acute sinusitis. ^3^Obstructive sleep apnea syndrome. ^4^Due to visual evaluation during intraoperative tympanocentesis and suction. ^5^A: normal curve; B: flat curve due to middle ear fluid. C: deviated curve due to negative middle ear pressure. All tympanometric measurements were performed the day before adenoidectomy. ^6^Recurring acute otitis media group. ^7^Otitis media with effusion group. ^8^Obstructive sleep apnea syndrome group.

**Table 2:** Individual pattern of respiratory virus specific real-time (RT) PCR.

Patient number	Virus-specific real-time PCR							
	Infl A^1^	Infl B^2^	RSV^3^	hMPV^4^	RV/EV^5^	AV^6^	hBoV^7^	CC^8^
1	−	−	−	−	+	+	−	+
2	−	−	−	−	+	+	+	+
3	−	−	−	−	+	−	−	+
4	−	−	−	−	+	−	−	+
5	−	−	−	−	+	+	−	+
6	−	−	−	−	−	+	+	+
7	−	−	−	−	+	−	−	+
8	−	−	−	−	−	−	−	+
9	−	−	−	−	+	+	−	+
10	−	−	−	−	+	+	−	+
11	−	−	−	−	−	+	−	+
12	−	−	−	−	−	+	−	+
13	−	−	−	−	+	−	−	+
14	−	−	−	−	+	+	−	+
15	−	−	−	−	−	−	+	+
16	−	−	−	−	−	+	+	+
17	−	−	−	−	−	+	+	+
18	−	−	−	−	−	+	−	+
19	−	−	−	−	+	+	+	+
20	−	−	−	−	+	+	−	+
21	−	−	−	−	−	−	+	+
22	−	−	−	−	+	−	+	+
23	−	−	−	−	−	−	+	+
24	−	−	−	−	+	+	−	+
25	−	−	−	−	+	−	+	+
26	−	−	−	+	−	−	+	+
27	−	−	−	−	+	−	−	+
28	−	−	−	−	−	−	−	+
29	−	−	−	−	+	−	+	+
30	−	−	−	−	+	+	+	+
31	−	−	−	−	−	−	+	+
32	−	−	−	−	−	−	+	+
33	−	−	−	−	−	+	−	+
34	−	−	−	−	−	+	−	+
35	−	−	−	−	+	+	−	+
36	−	−	−	−	−	−	+	+
37	−	−	−	−	−	+	−	+
38	−	−	−	−	−	+	+	+
39	−	−	−	−	−	+	−	+
40	−	−	−	−	−	−	−	+
41	−	−	−	−	−	+	−	+
42	−	−	−	−	−	+	+	+
43	−	−	−	−	−	−	−	+
44	−	−	−	−	−	−	+	+
45	−	−	−	−	−	+	−	+
46	−	−	−	−	−	−	+	+
47	−	−	+	−	+	−	+	+
48	−	−	−	−	−	−	−	+
49	−	−	−	−	−	−	−	+
50	−	−	−	−	+	+	+	+
51	−	−	−	−	−	−	−	+
52	−	−	−	−	−	−	+	+
53	−	−	−	−	−	+	−	+
54	−	−	−	−	+	+	+	+
55	−	−	−	−	+	+	−	+
56	−	−	−	−	−	−	+	+
57	−	−	−	+	−	−	+	+
58	−	−	−	−	−	−	+	+
59	−	−	−	−	+	+	+	+

^1^Influenza-A virus, ^2^influenza-B virus, ^3^respiratory syncytial virus, ^4^human metapneumovirus, ^5^rhinovirus and enterovirus, ^6^adenovirus, ^7^human bocavirus, and ^8^cellular control.

**Table 3:** Summary of respiratory virus specific real-time (RT) PCR results and histologic findings in different patient groups.

	Patient groups (*n* = 59)		
	RAOM group (*n* = 25)	OME group (*n* = 19)	OSAS group (*n* = 15)
*Real-time PCR *			
Infl A	0	0	0
Infl B	0	0	0
RSV	0	1	0
hMPV	0	2	0
RV/EV	8	7	11
AV	10	7	8
hBoV	0	19	8
*Cellular control *	25	19	15
*Biofilm positivity *	20	0	1
*Mucosa *			
Normal respiratory mucosa	5	19	15
Mucosal disintegration and/or metaplasia	20	0	0
*Stroma *			
Normal structure with centrum germinativum	8	19	15
Neutrophil and/or eosinophil granulocyte infiltration	17	0	0

**Table 4:** Respiratory virus coexpression pattern in different patient groups.

Virus co-expression pattern	Patient groups (*n* = 59)		
	RAOM group (*n* = 25)	OME group (*n* = 19)	OSAS group (*n* = 15)
Undetectable	7 (28%)	0	0
RV/EV	3 (12%)	0	3 (20%)
RV/EV + AV	5 (20%)	0	4 (27%)
RV/EV + hBoV	0	1 (5%)	3 (20%)
RV/EV + AV + hBoV	0	5 (26%)	1 (7%)
RV/EV + RSV + hBoV	0	1 (5%)	0
AV	10 (40%)	0	0
hBoV	0	8 (42%)	1 (7%)
AV + hBoV	0	2 (10%)	3 (20%)
hMPV + hBoV	0	2 (10%)	0
